# Chronic cortisol differentially impacts stem cell-derived astrocytes from major depressive disorder patients

**DOI:** 10.1038/s41398-021-01733-9

**Published:** 2021-11-30

**Authors:** Kelly J. Heard, Maxim N. Shokhirev, Caroline Becronis, Callie Fredlender, Nadia Zahid, Amy T. Le, Yuan Ji, Michelle Skime, Timothy Nelson, Daniel Hall-Flavin, Richard Weinshilboum, Fred H. Gage, Krishna C. Vadodaria

**Affiliations:** 1 Laboratory of Genetics, The Salk Institute for Biological Studies, 10010 North Torrey Pines Road, La Jolla, CA 92037 USA; 2 The Razavi Newman Integrative Genomics and Bioinformatics Core (IGC), The Salk Institute for Biological Studies, 10010 North Torrey Pines Road, La Jolla, CA USA; 3 Department of Molecular Pharmacology and Experimental Therapeutics, Mayo Clinic, Rochester, MN USA; 4 University of Utah School of Medicine, Salt Lake City, UT USA; 5 Department of Psychiatry and Psychology, Mayo Clinic, Rochester, MN USA

**Keywords:** Molecular neuroscience, Depression

## Abstract

Major depressive disorder (MDD) is a prevalent psychiatric disorder, and exposure to stress is a robust risk factor for MDD. Clinical data and rodent models have indicated the negative impact of chronic exposure to stress-induced hormones like cortisol on brain volume, memory, and cell metabolism. However, the cellular and transcriptomic changes that occur in the brain after prolonged exposure to cortisol are less understood. Furthermore, the astrocyte-specific contribution to cortisol-induced neuropathology remains understudied. Here, we have developed an in vitro model of “chronic stress” using human induced pluripotent stem cell (iPSC)-derived astrocytes treated with cortisol for 7 days. Whole transcriptome sequencing reveals differentially expressed genes (DEGs) uniquely regulated in chronic cortisol compared to acute cortisol treatment. Utilizing this paradigm, we examined the stress response transcriptome of astrocytes generated from MDD patient iPSCs. The MDD-specific DEGs are related to GPCR ligand binding, synaptic signaling, and ion homeostasis. Together, these data highlight the unique role astrocytes play in the central nervous system and present interesting genes for future study into the relationship between chronic stress and MDD.

## Introduction

Physiological stress is the body’s way of responding to potentially dangerous stimuli, both internal and external. Stress activates the hypothalamic-pituitary-adrenal axis (HPA-axis) and elicits a well-characterized response to respond to the stressor and return bodily function to homeostasis [1]. Glucocorticoids, the main output of the HPA-axis, have receptors that, when activated, translocate to the nucleus and bind DNA, regulating expression of target genes [2]. This transcriptional regulation can have vastly different effects depending on tissue and cell type, including redirecting metabolic energy stores, regulating vasoconstriction in peripheral tissue, dilating pulmonary blood vessels, and promoting adaptive responses in the brain [3]. In the short term, responding to a stressor can be beneficial, but prolonged exposure to stressors, deemed “chronic stress,” disrupts feedback regulation of the HPA-axis and the natural cycling of glucocorticoids. Chronic stress can lead to immunosuppression, cardiovascular disease, osteoporosis, neurodegeneration, and other health risks [3].

Chronic stress and disruption of the HPA-axis accompany many psychiatric disorders, including major depressive disorder (MDD) [4, 5], anxiety disorder, and bipolar disorder [6, 7]. MDD is a leading cause of psychiatric disability, with over 250 million cases worldwide [8]. Because it is a heterogeneous disorder, it has proven difficult to determine its biological underpinnings or identify a universally effective treatment. Clinical research of chronic stress in MDD patients, reviewed extensively by Nandam et al [6], has shown that HPA-axis disfunction is commonly found in depressed patients, and circulating stress hormone levels are affected by antidepressant treatment. Interestingly, elevated serum and salivary cortisol levels are seen most consistently in a subset of patients with severe depression, as opposed to mild and atypical depression [6]. While increased cortisol concentration is a simplistic explanation of chronic stress and is not a universal biomarker of MDD, it is clear from pre-clinical studies that chronic exposure to increased cortisol confers pathological susceptibility at least in a subset of MDD patients.

Decades of research in rodent models and clinical studies have revealed some of cortisol’s effect on neurons, including reduction of adult neurogenesis, decreased brain volume, and impaired learning and memory [9]. Recently, glial cells such as astrocytes have come to the forefront of research into central nervous system function. With end feet surrounding synapses and blood vessels, astrocytes tile every region of the brain, regulating ion homeostasis, neurotransmitter pools, synaptic plasticity, and neuronal support [10, 11]. Despite their important role in the brain, astrocytes have a myriad of functions that are understudied with respect to their role in response to stress and their role in MDD. Glucocorticoids have been shown to disrupt cell junctions that are necessary between astrocytes to shuttle metabolites such as lactate and glucose [12]. Uncoupling these cell junctions may contribute to learning and memory deficits observed in chronic stress animal models [13]. Furthermore, astrocytes are immunoreactive and may interact with the HPA-axis through secreted cytokines such as IL-1 and IL-6 [14]. While some cellular phenotypes have been identified, the broad level impact of chronic glucocorticoids exposure on astrocytes is unclear.

In this study, we have developed an in vitro model of chronic cortisol exposure to investigate the key transcriptomic changes that occur in human astrocytes and use that paradigm to investigate the specific impact of “chronic stress” in MDD patient-derived astrocytes. Using a protocol we previously developed, we derived astrocytes from human induced pluripotent stem cells (iPSCs) and optimized a 7-day exposure paradigm vs. acute exposure on a single day. Whole transcriptome analysis revealed unique changes to genes involved with cell adhesion, extracellular matrix organization, and cell morphogenesis. Using the 7-day exposure paradigm, we studied the specific impact of chronic cortisol exposure in MDD patient-derived astrocytes with whole transcriptome analyses. We discovered uniquely disrupted pathways in MDD astrocytes exposed to chronic cortisol. Genes involved in GPCR ligand binding, ion transport, and synaptic transmission were uniquely affected by chronic stress in MDD astrocytes, notably the serotonin receptor HTR1B, the neurotrophic factor GDNF, and glutamate receptors GRM1 and GRM8. We have generated a novel in vitro chronic stress-like paradigm and provide evidence for the unique regulation of astrocyte genes in MDD. Further, the whole transcriptome data set from a total of 9 human iPSC-derived astrocytes provide a rich and unbiased data set for future study into the molecular mechanisms of stress and its role in exacerbating psychiatric disorders.

## Materials and methods

### Cell culture

Fibroblasts were derived from skin punch biopsies and converted to iPSCs by virally introducing reprogramming factors SOX2, KLF9, OCT3/4, and c-Myc as previously described [15]. Glial progenitor cells (GPCs) were derived from iPSCs as previously described [16]. Briefly, iPSC colonies were lifted enzymatically, transferred to ultra-low-attachment plates, and placed on an orbital shaker for 3 weeks as embryoid bodies. Embryoid bodies were dissociated into GPCs and passaged 4–6 times to eliminate neuronal cells. GPCs were differentiated on Matrigel-coated plates in serum-free differentiation media for 4 weeks. Serum-free differentiation media contained DMEM F12 + Glutamax (Thermo Fisher; Waltham, MA), 10% KOSR (Thermo Fisher), N-2 supplement (Thermo Fisher), 20 ng/mL CNTF (Proteintech; Rosemont, IL), 20 ng/mL BMP4 (PeproTech; Rocky Hill, NJ), 20 ng/mL FGF2 (Joint Protein Central, Korea), and 20 ng/mL EGF (Humanzyme; Chicago, IL). All cell lines were regularly tested for mycoplasma contamination.

### Cortisol treatment

Cortisol (Sigma Aldrich) was reconstituted in Ethanol (EtOH) to a stock concentration of 100 mM kept at -20 °C. Cells were treated with 50 µM cortisol (0.005% EtOH) or 5 µM cortisol (0.0005% EtOH) for acute treatment. Chronic cortisol-treated cells received 5 µM cortisol or 0.0005% EtOH every 48 h for 7 days and were collected 24 h after the last dose.

### Patient information

Patient iPSCs were derived from a cohort of patients previously described [17] that were chosen from a larger pharmacogenetic study [18]. Briefly, all patients were adult females (ages 33 to 53) with depression scored via HAMD-17 and QIDS-C16 rating scales. After discussing the risks and benefits of the study, all participants provided written informed consent. Skin punch biopsies from patients were collected in sterile conditions, and all procedures were overseen by the Institutional Review Board of the Mayo Clinic as previously described [17].

### Immunocytochemistry

GPCs and astrocytes were fixed with 4% paraformaldehyde for 15 min at 4 °C. Fixed cells were washed before 30-min incubation at room temperature with blocking solution: 0.1% Triton X-100 and 3% normal horse serum in PBS. Cells were incubated with primary antibodies in blocking solution at 4 °C overnight. Primary antibodies used were rat anti-CD44 (1:500; BD Pharmingen 550538, San Diego, CA), rabbit anti-NF1-A (1:250; Novus Biologicals NBP1-81406, Centennial, CO), mouse IgM anti-A2B5 (1:100; EMD Millipore MAB312, Burlington, MA), rabbit anti-S100β (1:1000; Dako GA504, Santa Clara, CA), goat anti-Vimentin (1:500; EMD Millipore AB1620), rabbit anti-DCX (1:1000; Cell Signaling 14802S, Danvers, MA), chicken anti-MAP2ab (1:1000; Abcam ab5392, United Kingdom). Cells were washed before incubation with secondary antibodies in blocking solution at 4 °C for 1 h at room temperature. Nuclei were visualized with DAPI and slides were coverslipped with polyvinyl alcohol DABCO mounting medium (Sigma Aldrich, St Louis, MO).

### Microscopy

Bright field images were taken on an Olympus IX51 inverted microscope at 4x, 10x and 20x magnification. Fluorescent images were taken on a Zeiss Observer Z1 inverted microscope at 20x magnification.

### RNA extraction

Bulk RNA was collected using TriZol (Invitrogen; Carlsbad, CA) and kept at −80 °C before extraction. Briefly, chloroform was incubated with TriZol samples for 10 min on ice. Samples were centrifuged at 12,000 rcf for 15 min at 4 °C. The clear liquid phase was transferred to a new tube with isopropanol and incubated for 10 min at room temperature. Precipitate was pelleted with centrifugation at 12,000 rcf for 10 min at 4 °C. The pellet was washed with cold 75% EtOH followed by 100% EtOH before removing liquid and drying at room temperature for 20 min. The dry pellet was dissolved in 20 uL nuclease-free water.

### Semi-quantitative real time PCR

The extracted 5 µg RNA was treated with DNAse (Life Technologies; Carlsbad, CA) per manufacturer’s instructions. Samples were reverse transcribed using SuperScript III cDNA kit (Thermo Fisher) per manufacturer’s instructions. PCR reaction was run in triplicate for each sample with SYBR Green master mix (Thermo Fisher) and 500 nM of each primer. Relative expression for each sample was determined by normalizing cycle threshold (Ct) of the gene of interest to the Ct of the housekeeping gene ACTB via subtraction (dCt), subtracting dCt of cortisol treatment from dCt of its own vehicle control (ddCt), and calculating fold change. Primer sequences are provided in Supplementary Table 1.

### Data analysis

Experiments were repeated as independent triplicates to obtain averages per individual per group for further statistical analysis. When comparing groups, statistical comparisons were performed by comparing average values from individuals per group. RNA-sequencing data was analyzed with the statistical package built into the DeSeq2 package.

### RNA-sequencing analysis

Sequenced reads were quality-tested using FASTQC [19] v0.11.8 and aligned to the hg19 [20] human genome using the STAR aligner [21] version 2.5.3a. Mapping was carried out using default parameters, filtering non-canonical introns and allowing up to 10 mismatches per read and only keeping uniquely mapped reads. The genome index was constructed using the gene annotation supplied with the hg19 Illumina iGenomes [22] collection and sjdbOverhang value of 100. When necessary, Illumina barcodes were trimmed using trimgalore [23]. Raw or TPM (transcripts per million) gene expression was quantified across all gene exons with HOMER [24] using the top-expressed isoform as proxy for gene expression, and differential gene expression was carried out on the raw counts using the DeSeq2 [25] package version 1.22.2 using duplicates to compute within-group dispersion and treating cell-line identity as a batch variable to account for cell-line technical bias. Differentially expressed genes (DEGs) were defined as having a false discovery rate (FDR) < 0.1 and a log2 fold change >0.585 (~1.5 fold) when comparing 2 experimental conditions. Principal component analysis (PCA) was carried out using the prcomp R function on the top 2000 expressed TPM gene values. Overrepresentation analysis was carried out using MetaScape [26] with default parameters. We looked for significant overrepresentation of Gene Ontology (GO) biology process [27], Reactome [28], and MSigDB [29] Canonical Pathways. All sequencing data have been uploaded to NIH GEO data repository and can be accessed with the following token: GSE178071.

## Results

### Generating human iPSC-derived astrocytes under serum-free conditions

To study the effects of chronic stress on human astrocytes, we first optimized a serum-free in vitro model of iPSC-derived astrocytes. Reprogramming somatic cells to a pluripotent state allows us to investigate uniquely human biology and manipulate human cells in a dish while understanding the donor’s overall health. Typically, fetal bovine serum is used in vitro to provide nutrients necessary for cell growth. However, many studies have shown that each batch of serum has varying levels of growth factors and hormones, including glucocorticoids, which could interfere with our results [30]. To control for the concentration of stress hormones, we sought to adapt our previously published method to generate astrocytes with a defined culture medium free of any serum.

**Fig. 1:**
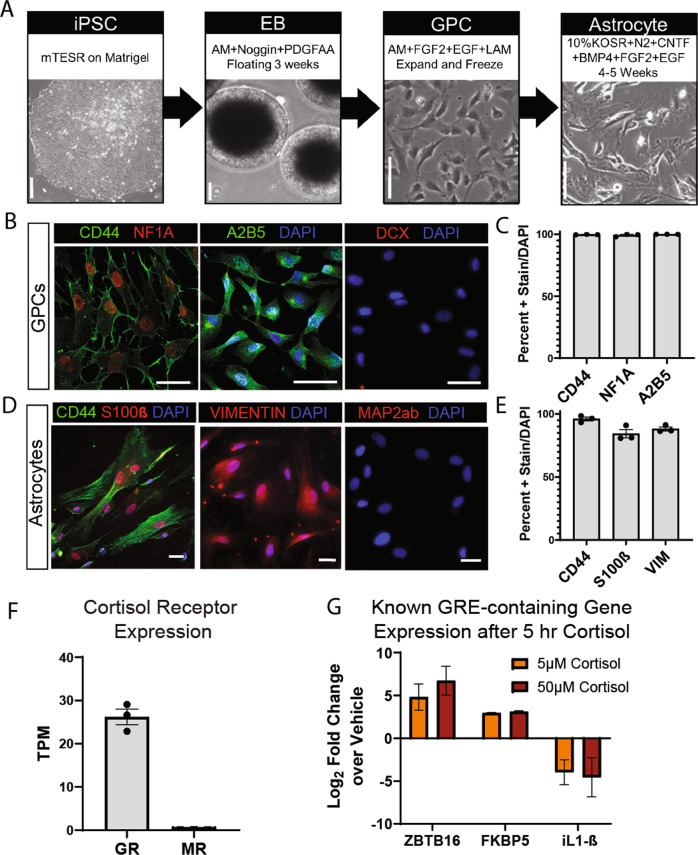
iPSC-derived astrocytes differentiated in serum-free conditions express appropriate glial markers and respond to cortisol in vitro. **a** Schematic of serum-free astrocyte differentiation from iPSCs via floating embryoid body (EB) and glial progenitor cell (GPC) intermediate stages. Representative bright field images depict major steps. Scale bars 100 µm. **b** Representative fluorescent images of immunostained GPCs expressing early glial fate markers CD44 (left-green), NF1A (left-red), and A2B5 (middle-green) and negative stain for early neuronal marker DCX (right-red); scale bars 50 µm. **c** Quantification of immunostain showing percent of GPCs expressing CD44, NF1A, and A2B5 out of DAPI identified total cells. Bars depict mean ± SEM; *n* = 3 individuals. **d** Representative fluorescent images of immunostained astrocytes expressing CD44 (left-green), S100β (left-red), and Vimentin (middle-red) and negative stain for neuronal marker Map2ab (red-right); scale bars 50 µm. **e** Quantification of immunostain showing percent of astrocytes expressing CD44, S100β, and Vimentin out of DAPI identified total cells. Bars depict mean ± SEM; *n* = 3 individuals. **f** Expression of cortisol receptors Glucocorticoid Receptor (GR) and Mineralocorticoid Receptor (MR) measured via whole transcriptome sequencing under baseline conditions. Bars show mean TPM ± SEM; *n* = 3 individuals. **g** Expression of known Glucocorticoid Response Element (GRE)-containing genes, ZBTB16, FKBP5, and L-1β, following 5 h of Cortisol or Vehicle treatment at 5 µM or 50 µM concentration. RT-qPCR values expressed as log_2_fold change over own vehicle control; bars depict mean ± SEM; *n* = 6 with 3 replicates in 2 individuals.

We derived iPSCs from the fibroblasts of 3 healthy individuals and followed our previously published protocol [16] to generate glial progenitor cells (GPCs) (Fig. 1A). At this stage, all lines (~99–100% of cells) express early glial fate markers CD44, Nuclear Factor 1A (NF1-A), and A2B5 (Fig. 1B,C). The expression of these markers indicates that we have a homogeneous population of cells with glial differentiation capacity. To convert GPCs to astrocytes, we utilized a defined medium that relies on Knock-out Serum (KOSR), N-2 supplement, Fibroblast Growth Factor 2 (FGF2), and Epidermal Growth Factor (EGF) to support cell growth in combination with Ciliary Neurotrophic Factor (CNTF) and Bone Morphogenic Protein 4 (BMP4) to specify astrocyte fate (Fig. 1A). Astrocytes differentiated for 4 weeks expressed (~90–100% of cells) astrocyte markers CD44, S100β, and Vimentin (Fig. 1D,E). The expression of these makers was comparable to our previous work, suggesting that serum-free conditions did not affect the differentiation capacity of iPSC-derived astrocytes. Furthermore, GPC and astrocyte cultures co-stained with early neuronal markers Doublecortin (DCX) and Microtubule Associated Protein 2ab (MAP2ab), respectively, showed that there were virtually no neurons in the culture (Fig. 1B, D). These results showed that we had derived a homogeneous population of cells expressing typical markers of astrocytic fate. The high purity of these cultures is likely the result of a combination of glial patterning factors and the fact that lingering neural progenitors would not survive passaging and differentiation in the astrocyte culture medium used.

We wanted to further validate serum free-raised astrocytes by investigating astrocyte function. Glutamate transporter EAAT2 is necessary for astrocytes to traffic neuronal glutamate in vivo. At 4 weeks of differentiation, we found that serum-free astrocytes expressed EAAT2 (Supplementary Fig. 1A), indicating our cells had the capacity for this key astrocytic process. Astrocytes also become activated in response to inflammatory stimuli, secreting cytokines and contributing to neuroinflammatory response in vivo. To determine the capability of serum-free astrocytes to become activated, we treated cells with inflammatory cytokine iL1-β and found that astrocytes produced a multitude of pro-inflammatory cytokines (Supplementary Fig. 1B,C). Furthermore, ~25–60% of cells produced IL-6, and ~55–80% of cells produced IL-8 following activation (Supplementary Fig. 1D,E). These data indicate that astrocytes grown in serum-free conditions are able to mount an inflammatory response, and the percentage of activated cells is comparable to percentages we see in astrocytes differentiated with serum [16]. Together, these results validate our model, showing that serum-free astrocytes express appropriate differentiation markers and possess functional properties important for astrocyte cellular function.

### Human iPSC-derived astrocytes respond to cortisol in vitro

To investigate how chronic stress impacts astrocytes in the brain, we first wanted to examine if iPSC-derived astrocytes responded to cortisol in vitro. Cortisol binds to two receptors: low-affinity Glucocorticoid Receptor (GR) and high-affinity Mineralocorticoid Receptor (MR). When bound to its ligand, GR and MR translocate to the nucleus and bind to specific sequences of DNA deemed Glucocorticoid Response Elements (GRE) to enact transcriptional changes [1]. We examined receptor expression under baseline conditions and found that iPSC-derived astrocytes robustly expressed GR and did not express MR (Fig. 1F). These results indicate that GR may mediate a response to cortisol in these cells, whereas MR is negligible in our analysis.

We next examined if cortisol treatment in vitro could elicit transcriptional changes through GR at this stage of differentiation. While blood and salivary cortisol measurements in vivo rarely exceed 1 uM, most in vitro studies of stress range from 500 nM to 100 uM [31]. We treated astrocytes for 5 h with 2 concentrations of cortisol, 5 and 50 µM, or their corresponding percentage of ethanol as a vehicle control. Given the acute time frame, we looked at expression of immediate response genes that contained a GRE and were typically dysregulated after cortisol treatment. Using qPCR, we observed that cortisol increased expression of cell cycle-related transcription factor ZBTB16 (log_2_fold change = 4.82 at low dose and 6.73 at high dose) and stress response modulator FKBP5 (log_2_fold change = 2.97 at low dose and 3.10 at high dose) compared to vehicle. In addition, cortisol decreased expression of pro-inflammatory cytokine iL1-β (log_2_fold change = −3.97 at low dose and −4.56 at high dose) (Fig. 1G). These results suggested that the GR was functionally active in human iPSC-derived astrocytes in vitro, responded to cortisol and transcriptionally regulated known targets. Fold change between 5 and 50 µM concentrations of cortisol was comparable, hence we proceeded to use the lower dose (5 µM) cortisol for the rest of the study to better relate to concentrations found in vivo.

### Chronic vs acute cortisol treatment elicits transcriptional changes in human astrocytes

**Fig. 2:**
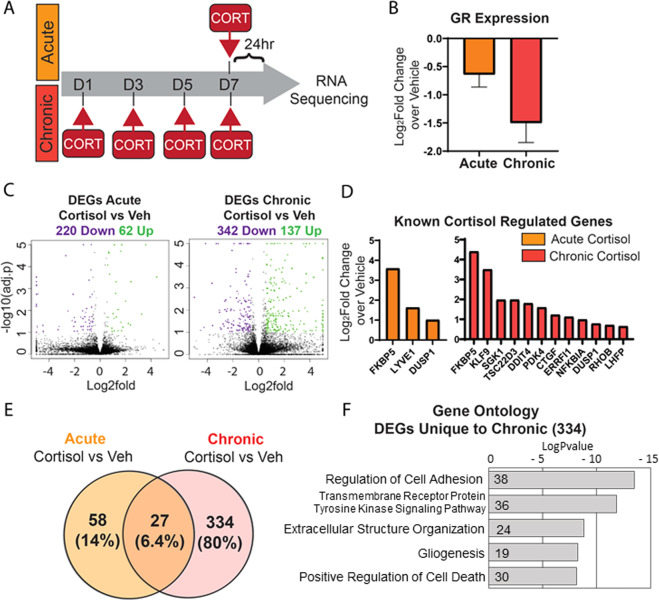
Chronic cortisol treatment in astrocytes has a unique transcriptomic signature present 24 h after last dose. **a** Schematic of acute vs chronic cortisol treatment experimental paradigm. Under chronic conditions, astrocytes are treated with 5 µM cortisol or vehicle every 48 h for 7 days and collected for experiments 24 h after the last dose. In the acute condition, astrocytes are given one treatment 24 h before collection. **b** Expression of GR following acute or chronic cortisol treatment measured via RT-qPCR. Log_2_fold change over own vehicle control graphed as mean ± SEM; *n* = 6 with 3 replicates in 2 individuals. **c** Volcano plots depicting differentially expressed genes (DEGs) upregulated (green dots) or downregulated (purple dots) following treatment with cortisol; *n* = 2 individuals. **d** Expression of genes known to be regulated by cortisol in vivo that are DEGs in our samples. Expression represented as log_2_fold change over vehicle. **e** Venn diagram comparison of the number of DEGs unique to each treatment and those that overlap. **F** Top categories from Gene Ontology (GO) analysis of DEGs unique to chronic cortisol (334 genes); bars represent the log *P* value of the term and numbers within the bar represent the genes in our list under that term.

Given that chronic and not acute stress is a risk factor for MDD, we sought to generate a model of chronic cortisol exposure. To compare the transcriptional profiles of chronic vs. acute exposure, we developed a 7-day cortisol treatment paradigm depicted in Fig. 2A. Astrocytes were treated every 48 h for 7 days with either 5 µM cortisol or ethanol vehicle control and cells were harvested for RNA collection 24 h after the last dose. An acute cortisol treatment was run in parallel, treating astrocytes for 24 h before RNA collection (Fig. 2A). We performed RNA-sequencing analysis and found several DEGs, as listed in Supplementary Tables 2 and 3. First, we wanted to determine the effect of ethanol as a vehicle on the cells. Direct comparison between acute and chronic vehicle treatment revealed only 1 DEG (Supplementary Fig. 2A), indicating that the effect of a minimal concentration of ethanol as a vehicle for these experiments was minimal (Supplementary Fig. 2A). It is well known that GR downregulates its own expression, thereby tempering the cell’s response to stress [32]. To determine if our model elicited this form of negative feedback, we examined GR expression via qPCR and found that it was reduced following cortisol exposure both acutely and chronically (Fig. 2B). These data induced confidence that our treatment paradigm recapitulated the complex downstream effects of cortisol and feedback regulation in our iPSC-derived astrocytes.

We next compared the DEGs in the acute and chronic treatment paradigms. Acute cortisol compared to its own vehicle resulted in 62 upregulated genes and 220 downregulated genes, whereas chronic cortisol compared to its own vehicle resulted in 137 upregulated genes and 342 downregulated genes. Firstly, we noted that chronic treatment dysregulated nearly double (~1.7) the number of genes as compared to acute treatment (compared to their respective control groups) (Fig. 2C), highlighting a unique set of genes dysregulated only with chronic cortisol treatment. To further validate our model, we compared these DEGs with previous work in the glucocorticoid field. Using a compiled list of 88 overlapping genes from 21 studies both in vivo and in vitro [31], we found several genes that were similarly regulated in our model. Notably, acute treatment upregulated 3 genes, FKBP5, LYVE1, and DUSP1, whereas chronic treatment upregulated 12 of these genes (Fig. 2D). This result corroborated our findings and suggested that the data found in vitro might be relevant to understanding astrocyte-specific aspects of stress response in humans.

Next, to better understand the impact of chronic treatment on human astrocytes, we examined the unique set of DEGs only regulated with chronic treatment. A total of 334 genes are dysregulated specifically after 7 days of chronic cortisol treatment, whereas 58 genes are unique to acute treatment and 27 genes appear in both treatment lists (Fig. 2E and Supplementary Table 4). To better understand how these DEGs relate to cellular processes, we used Gene Ontology (GO) overrepresentation analysis, which compares a user’s list of genes to a large database of biomedical terms. The enrichment analysis provides insight into which pathways and protein complexes are overrepresented among the genes of interest [26]. Analysis of the 334 genes unique to chronic cortisol returned the following GO terms: *regulation of cell adhesion, tyrosine kinase signaling, extracellular matrix organization, gliogenesis*, and *positive regulation of cell death* (Fig. 2F and Supplementary Table 5). This suggests that major pathways involved in intracellular morphology as well as extracellular environment were affected only following chronic cortisol.

### MDD astrocytes regulate a unique set of genes in response to chronic cortisol

We next wanted to investigate the relationship between chronic stress and MDD. We examined the DEG list unique to chronic cortisol and found 2 genes that had been previously implicated in the disease: Monoamine oxidase A (MAOA) (Log_2_Fold Change = 2.59) and Neurotrophic Tyrosine Kinase Receptor 2 (NTRK2) (Log_2_Fold Change = −1.15). MAOA is responsible for the metabolism of monoamine neurotransmitters such as serotonin, dopamine, and norepinephrine, and polymorphisms in the promoter region of this gene have been associated with MDD pathology [33]. NTRK2, also known as TrkB, and its ligand Brain-derived neurotrophic factor (BDNF) are critical for neuronal survival and differentiation. Mutation and lower expression of NTRK2 have been observed in MDD patients [34]. The dysregulation of these genes in our model system highlights the relevance of these findings to the understanding of chronic stress in humans and suggests that the relationship between chronic stress and MDD can be meaningfully studied at a transcriptional level in this model.

**Fig. 3:**
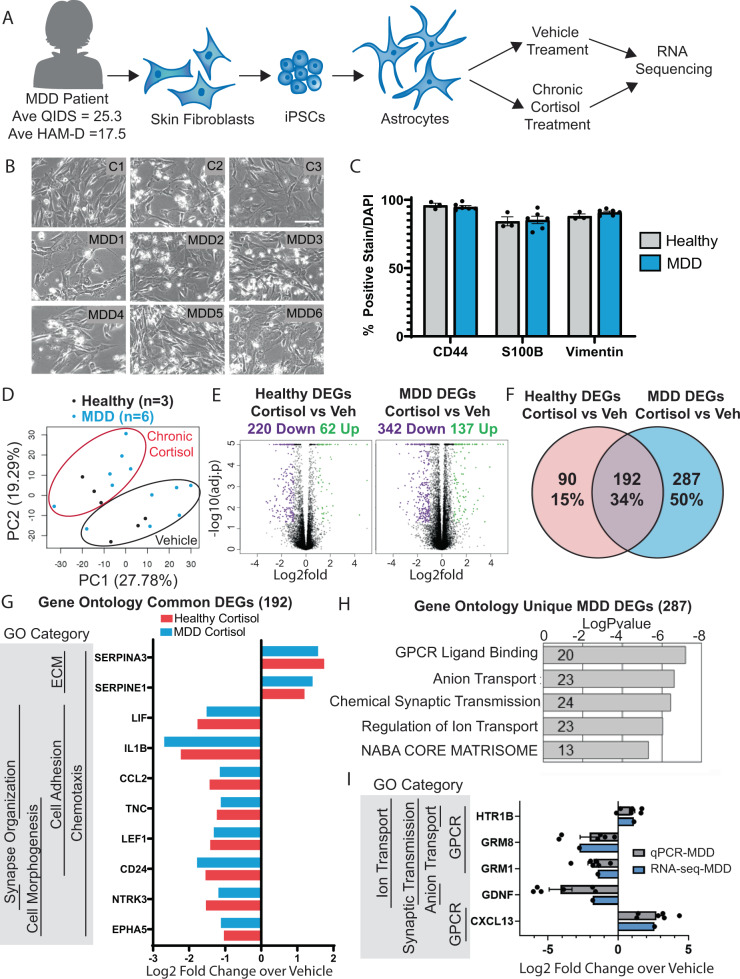
Chronic cortisol treatment elicts a unique transcriptional response in astrocytes derived from patients with MDD. **a** Schematic showing the process of deriving astrocytes from Major Depression (MDD) patient skin samples and treatment with chronic cortisol for RNA-sequencing. **b** Bright field representative images of astrocytes differentiated from 3 healthy control individuals and 6 MDD patients; scale bar = 100 µm. **c** Quantification of astrocyte marker expression measured via immunostain. Bars show mean ± SEM, *n* = 6 individuals (individuals are represented by dots). **d** Principal component analysis of transcriptome sequencing data in all treated samples; black dots = healthy individuals, blue dots = MDD individuals. **e** Volcano plots depicting differentially expressed genes (DEGs) upregulated (green dots) or downregulated (purple dots) following treatment with cortisol; *n* = 3 (Healthy); *n* = 6 (MDD). **f** Venn diagram comparison of DEGs unique to each cohort and those that overlap. **g** Selected highly significant genes regulated by chronic cortisol in both healthy and MDD individuals with top gene ontology categories, left. **h** Top gene ontology categories from DEGs unique to MDD (287 genes); bars represent the logPvalue of the term and numbers within the bar represent the genes in our list under that term. **i** Selected highly significant genes in top GO categories unique to MDD confirmed by RT-qPCR; bars show mean log_2_fold change over vehicle ± SEM for *n* = 6 (individuals are represented by dots).

While chronic stress is a robust risk factor of developing MDD, many individuals experience bouts of chronic stress and do not develop more severe psychiatric symptoms. It is clear that genetic background or early environmental cues predispose an individual, conferring differential stress susceptibility. Therefore, we hypothesized that astrocytes derived from patients with MDD might have a unique transcriptional response to our model of chronic stress. To explore this hypothesis we generated astrocytes from 6 patients with severe MDD (Fig. 3A). As part of a larger study, depression scores (average QIDS = 25.3 and HAM-D = 17.5) were used to identify and collect biopsies from patients with severe MDD (Supplementary Fig. 3B) as described previously [17]. We first compared efficacy of differentiation and functionality of MDD- and neurotypical individual (healthy)-derived astrocytes. Bright field images showed that MDD and healthy astrocytes were morphologically similar in the dish (Fig. 3B). Furthermore, astrocytes and GPCs from this cohort homogeneously expressed astrocyte differentiation markers (Fig. 3C; Supplementary Fig. 3A), indicating that astrocyte differentiation was comparable between the healthy and MDD lines. As a positive control and marker for differentiation, we confirmed that GR and EAAT2 were expressed in all lines (Supplementary Fig. 3C-D) and the response to inflammatory stimuli was similar between healthy and MDD lines (Supplementary Fig. 3E-G). These results showed that iPSC-derived astrocytes from MDD and healthy individuals were comparable in terms of differentiation capacity and selected cellular functions.

Next, we compared the transcriptome of MDD and healthy astrocytes under baseline conditions. RNA-sequencing revealed a small list of genes (20) differentially regulated in MDD compared to control (Supplementary Fig. 3H). Notably, expression of Metalloproteinase 9 (MMP9) and Connexin 26 (GJB2) were reduced in MDD astrocytes. Interestingly, MMP9 has been previously been associated with depression and increase in response to antidepressant treatments [35]. To further analyze these genes, we performed GO analysis and the terms enriched in our list of 20 genes included *regulation of cell adhesion* and *axon guidance* as top hits (Supplementary Fig. 3I). Interestingly, no genes or GO terms related to stress or response to glucocorticoids were identified. While there are likely transcriptional differences in MDD and healthy astrocytes in humans, it is not unexpected that we detected few baseline differences between groups given the small sample size (*n* = 3 healthy and *n* = 6 MDD patients) and the genetic variability in human samples. The lack of major transcriptional differences between groups under baseline conditions suggested at least that differentiation and culture conditions for individual-derived lines was similar between the groups. Notably, within these constraints, we found hundreds of dysregulated genes only after chronic cortisol treatment.

Next, we tested the hypothesis that chronic cortisol might elicit a unique response in MDD astrocytes. We treated both healthy and MDD lines with chronic cortisol or vehicle and collected RNA for RNA-sequencing. First, we used principal component analysis (PCA) to compare the overall transcriptome of each sample. PCA allows us to plot samples across uncorrelated variables, reducing the dimensionality of such a large dataset while maximizing variance. PCA revealed that samples clustered by their treatment with cortisol or vehicle and not disease state (Fig. 3D), suggesting that cortisol treatment dominated the effect on the transcriptome as compared to disease status. To understand the dysregulated genes driving this differential clustering, we extracted DEGs from the chronic cortisol samples compared to their individual vehicle controls. Chronic cortisol regulated 280 genes in healthy astrocytes and 479 genes in MDD astrocytes (Fig. 3E, Supplementary Table 6 and 7). Comparing these lists, 192 genes were dysregulated by chronic cortisol in both diseased and healthy astrocytes (Fig. 3F and Supplementary Table 8). Furthermore, the magnitudes of change between healthy or MDD astrocytes of the 192 dysregulated genes were also similar. These data show that cortisol treatment regulated some transcriptional networks regardless of disease background. To better understand how these transcriptional changes affected cellular function, we performed GO analysis of the 192 genes and the top GO terms were *synapse organization, cell morphogenesis, cell adhesion, extracellular matrix organization*, and *chemotaxis* (Fig. 3G and Supplementary Table 9). These categories aligned with our previous experiment, suggesting that chronic cortisol exposure might alter astrocyte morphology, migration, and their extracellular environment.

In addition to the universal astrocyte response to chronic cortisol, we were interested in the unique changes in MDD astrocytes. There were 287 DEGs dysregulated after chronic cortisol treatment only in MDD astrocytes (Fig. 3F). The MDD patients in this study were originally selected for their response to the SSRI (selective serotonin reuptake inhibitor) Escitalopram, with 3 individuals responding to treatment and 3 individuals not responding to treatment [17]. Although the samples did not cluster based on their response to SSRI treatment (Supplementary Fig. 4A) in the PCA analysis we compared transcriptomic changes after chronic cortisol treatment within SSRI treatment response subgroups. There were 209 DEGs in responders and 273 DEGs in nonresponders, with a majority of the same DEGs dysregulated in both groups (Supplementary Fig. 4B and Supplementary Table 10–13). Analysis of the small number of unique DEGs in SSRI-responder/non-responders suffered from the caveat of an even smaller *n* per group. Hence we focused our analyses on the unique 287 DEGs dysregulated after chronic cortisol treatment only in MDD astrocytes.

We first examined if these genes had been identified previously in other studies of MDD. We used DisGeNET [36], which uses an integrated database of genes and variants from publicly available data to reveal human diseases associated with a given list of genes. Strikingly, the list of 287 DEGs that were uniquely regulated in MDD astrocytes after chronic cortisol returned top hits Unipolar Depression (25 genes; Log10(*P*) = −8.00) and Mood Disorders (19 genes; Lop10(*P*) = −5.10). This was an interesting finding considering the unbiased nature of this analysis and the fact that treatment of a single cell type has a transcriptional signature that aligns with other studies showing disruptions of similar pathways in mood disorders. To better understand the cellular processes affected in MDD astrocytes, we ran GO analysis on the 287 DEGs unique to MDD. Top GO terms included *GPCR binding, anion transport, chemical synaptic transmission*, and *regulation of ion transport* (Fig. 3H and Supplementary Table 14). Several of these categories suggested further modulation of the extracellular environment both in matrix structure and chemical components. Furthermore, GPCR binding and synaptic transmission are critical functions of neural cells mediated by astrocytes and their modulation is a core mechanism of MDD. To confirm disease-associated genes of interest, we selected highly significant genes that appeared in several categories and confirmed their mRNA fold change by performing semi-quantitative Real Time PCR (Fig. 3I). We also examine cell proliferation and cell death with chronic cortisol treatment and do not observe differences in cell death (Supplementary Fig. 5) suggesting that significant transcriptional differences do not arise from potential toxicity of chronic cortisol treatment. Taken together, these data sets revealed that MDD astrocytes had a differential response to chronic cortisol that might contribute to MDD pathology.

## Discussion

Herein we present an optimized in vitro model for chronic stress in iPSC-derived astrocytes. With a serum-free protocol, we generated a homogeneous and functional population of astrocytes and identified unique transcriptomic changes following chronic exposure to cortisol. It is worth noting that the 334 genes only present in the chronically treated group were regulated even 24 h after the last exposure, suggesting that the typically transient effect of GR activation was exacerbated by chronic treatment and that chronic cortisol might regulate novel targets not affected after one spike in concentration.

Molecular changes following cortisol exposure have been difficult to identify, as timing and dosage can lead to different DEGs identified and, in some cases, opposite directionality. In their review, Juszczak et al. combined gene lists from a variety of experimental paradigms with cortisol in the brain [31]. We found that many DEGs in our list were consistently regulated across multiple studies, including genes associated with cell death (DUSP1), glucose metabolism (PDK4), cell growth and metabolism (CTGF, ERRF1), and inflammatory response (NFK1B). Given our small sample size in comparing acute vs chronic treatment (*n* = 2 individuals), these data help corroborate our experimental paradigm that returned some hits that were expected.

GO term and transcription factor enrichment analysis of our unique list suggest that chronic cortisol affects cell adhesion and extracellular matrix organization. These terms suggest that astrocyte morphology and tiling, both of which are central to astrocyte function, may be disrupted during chronic stress. Furthermore, astrocytes are key mediators of neuroinflammation, and cortisol is a known anti-inflammatory. But, the specific action of cortisol in astrocytes to enact anti-inflammatory effects has not been studied extensively. In addition to reducing expression of pro-inflammatory cytokines such as IL1-β, cortisol may alter monocyte infiltration by modulating astrocytes at the blood brain barrier, altering cell adhesion and matrix proteins.

We expanded this paradigm for chronic stress to study the unique changes in MDD astrocytes compared to healthy controls. While only subgroups of MDD patients regularly exhibit high baseline concentrations of cortisol, this paradigm did return DEGs in our patient cohort that have previously been associated with unipolar depression and other mood disorders. Our data suggest that GPCRs are uniquely dysregulated in astrocytes following chronic stress, highlighting the importance of glutamate receptors GRM1 and GRM8 and serotonin receptor HTR1B. Regulation of these receptors in addition to several genes in the solute carrier, SLC, family (amino acid, ion, and glutamate transporters) suggests that the intra- and extracellular environment is altered following chronic stress. This likely impacts the ability of MDD astrocytes to regulate synaptic components and act as a reservoir for neuronal support molecules. In addition, downregulation of GDNF specifically in the MDD cohort suggests greater impact on neurotrophic support for development and function. Together, these findings provide transcriptomic evidence that MDD astrocytes are more susceptible to chronic stress, which may lead to more severe consequences. Remarkably, healthy astrocytes treated with chronic cortisol dysregulated genes previously linked with depression including MAOA, which modulates neurotransmitter levels, and neurotrophin signaling gene NTRK2/TrkB [37, 38]. Recent studies showed that astrocytes robustly expressed TrkB and responded to BDNF secreted by neurons [39, 40]. Furthermore, levels of TrkB expression have been shown to directly correlate with the amount of BDNF trafficked to the recipient cell [40]. Our data indicate dysregulation of these signaling key pathways in chronic cortisol treated astrocytes, highlighting their potential role the pathophysiology of MDD following stress exposure.

iPSC technology is a powerful way to model and manipulate human tissue in vitro while knowing patient symptomology and response to treatment in the clinic. However, a major caveat is the maturity of the cells, as their expression of differentiation markers most closely maps to fetal astrocytes and early development. Our astrocytes show some functional activity and our chronic cortisol DEGs are corroborated by previous studies at various stages of development, which provides some confidence in our results. There is also a need for human model systems to identify gene regulation specific to humans, especially investigating uniquely human diseases such as MDD. In addition, cortisol does have the ability to cross the placenta [41], and these data would likely be relevant to models of maternal stress and brain development.

Overall, chronic stress and the effects of cortisol in general can be difficult to decipher as the effects on cells seems to be specific to timing, dosage, and cell type as well as other intracellular conditions. The model we present could easily be extended to other iPSC-derived cell types to better understand the unique response and cell-cell interaction during chronic stress. The DEGs and pathways affected by chronic cortisol in healthy and MDD astrocytes that we identified provide numerous avenues for future study into the cellular and molecular mechanisms that mediate the impact of stress in the brain.

## Supplementary information

Supplementary Table 1

Supplementary Table 2

Supplementary Table 3

Supplementary Table 4

Supplementary Table 5

Supplementary Table 6

Supplementary Table 7

Supplementary Table 8

Supplementary Table 9

Supplementary Table 10

Supplementary Table 11

Supplementary Table 12

Supplementary Table 13

Supplementary Table 14

Supplementary Information

## References

[1] Lightman SL, Birnie MT, Conway-Campbell BL (2020). Dynamics of ACTH and cortisol secretion and implications for disease. Endocr Rev.

[2] Lupien SJ, Juster RP, Raymond C, Marin MF (2018). The effects of chronic stress on the human brain: from neurotoxicity, to vulnerability, to opportunity. Front Neuroendocrinol.

[3] Radley J, Morilak D, Viau V, Campeau S (2015). Chronic stress and brain plasticity: mechanisms underlying adaptive and maladaptive changes and implications for stress-related CNS disorders. Neurosci Biobehav Rev.

[4] Bertollo AG, Grolli RE, Plissari ME, Gasparin VA, Quevedo J, Réus GZ (2020). Stress and serum cortisol levels in major depressive disorder: a cross-sectional study. AIMS Neurosci.

[5] Ceruso A, Martínez-Cengotitabengoa M, Peters-Corbett A, Diaz-Gutierrez MJ, Martínez-Cengotitabengoa M (2020). Alterations of the HPA axis observed in patients with major depressive disorder and their relation to early life stress: a systematic review. Neuropsychobiology.

[6] Nandam LS, Brazel M, Zhou M, Jhaveri DJ (2020). Cortisol and major depressive disorder—translating findings from humans to animal models and back. Front Psychiatry.

[7] Keller J, Gomez R, Williams G, Lembke A, Lazzeroni L, Murphy GM (2017). HPA axis in major depression: cortisol, clinical symptomatology and genetic variation predict cognition. Mol Psychiatry.

[8] Depression. 2020. https://www.who.int/news-room/fact-sheets/detail/depression Accessed 7 Jan 2021.

[9] Nasca C, Bigio B, Zelli D, Nicoletti F, McEwen BS (2015). Mind the gap: glucocorticoids modulate hippocampal glutamate tone underlying individual differences in stress susceptibility. Mol Psychiatry.

[10] Allen NJ, Eroglu C (2017). Cell biology of astrocyte-synapse interactions. Neuron.

[11] Almad A, Maragakis NJ (2018). A stocked toolbox for understanding the role of astrocytes in disease. Nat Rev Neurol.

[12] Murphy-Royal C, Johnston AD, Boyce AKJ, Diaz-Castro B, Institoris A, Peringod G (2020). Stress gates an astrocytic energy reservoir to impair synaptic plasticity. Nat Commun.

[13] Pearson-Leary J, Osborne DM, McNay EC (2016). Role of glia in stress-induced enhancement and impairment of memory. Front Integr Neurosci.

[14] Luarte A, Cisternas P, Caviedes A, Batiz LF, Lafourcade C, Wyneken U, et al. Astrocytes at the hub of the stress response: potential modulation of neurogenesis by miRNAs in astrocyte-derived exosomes. *Stem Cells Int*. (2017) 10.1155/2017/1719050. 10.1155/2017/1719050 PMC5610870 29081809

[15] Mertens J, Wang QW, Kim Y, Yu DX, Pham S, Yang B (2015). Differential responses to lithium in hyperexcitable neurons from patients with bipolar disorder. Nature.

[16] Santos R, Vadodaria KC, Jaeger BN, Mei A, Lefcochilos-Fogelquist S, Mendes APD (2017). Differentiation of inflammation-responsive astrocytes from glial progenitors generated from human induced pluripotent stem cells. Stem Cell Rep.

[17] Vadodaria KC, Ji Y, Skime M, Paquola A, Nelson T, Hall-Flavin D (2019). Serotonin-induced hyperactivity in SSRI-resistant major depressive disorder patient-derived neurons. Mol Psychiatry.

[18] Mrazek DA, Biernacka JM, McAlpine DE, Benitez J, Karpyak VM, Williams MD (2014). Treatment outcomes of depression: the pharmacogenomic research network antidepressant medication pharmacogenomic study. J Clin Psychopharmacol.

[19] Babraham Bioinformatics - FastQC a quality control tool for high throughput sequence data. https://www.bioinformatics.babraham.ac.uk/projects/fastqc/ Accessed April 2021.

[20] Lander ES, Linton LM, Birren B, Nusbaum C, Zody MC, Baldwin J (2001). Initial sequencing and analysis of the human genome. Nature.

[21] Dobin A, Davis CA, Schlesinger F, Drenkow J, Zaleski C, Jha S (2013). STAR: Ultrafast universal RNA-seq aligner. Bioinformatics.

[22] Illumina. iGenomes Online. 2015. https://support.illumina.com/sequencing/sequencing_software/igenome.html Accessed April 2021.

[23] Felix K GitHub - FelixKrueger/TrimGalore: a wrapper around Cutadapt and FastQC to consistently apply adapter and quality trimming to FastQ files, with extra functionality for RRBS data. https://github.com/FelixKrueger/TrimGalore Accessed April 2021.

[24] Heinz S, Benner C, Spann N, Bertolino E, Lin YC, Laslo P (2010). Simple combinations of lineage-determining transcription factors prime cis-regulatory elements required for macrophage and B cell identities. Mol Cell.

[25] Love MI, Huber W, Anders S (2014). Moderated estimation of fold change and dispersion for RNA-seq data with DESeq2. Genome Biol.

[26] Zhou Y, Zhou B, Pache L, Chang M, Khodabakhshi AH, Tanaseichuk O (2019). Metascape provides a biologist-oriented resource for the analysis of systems-level datasets. Nat Commun.

[27] Carbon S, Douglass E, Good BM, Unni DR, Harris NL, Mungall CJ (2021). The gene ontology resource: enriching a GOld mine. Nucleic Acids Res.

[28] Jassal B, Matthews L, Viteri G, Gong C, Lorente P, Fabregat A (2020). The reactome pathway knowledgebase. Nucleic Acids Res.

[29] Subramanian A, Tamayo P, Mootha VK, Mukherjee S, Ebert BL, Gillette MA (2005). Gene set enrichment analysis: a knowledge-based approach for interpreting genome-wide expression profiles. Proc Natl Acad Sci USA.

[30] Van der Valk J (2018). Fetal Bovine Serum (FBS): past-present-future. ALTEX.

[31] Juszczak GR, Stankiewicz AM (2018). Glucocorticoids, genes and brain function. Prog Neuro-Psychopharmacol Biol Psychiatry.

[32] Okret S, Dong Y, Brönnegård M, Gustafsson JÅ (1991). Regulation of glucocorticoid receptor expression. Biochimie.

[33] Yu YW, Tsai SJ, Hong CJ, Chen TJ, Chen MC, Yang CW (2005). Association study of a Monoamine oxidase A gene promoter polymorphism with major depressive disorder and antidepressant response. Neuropsychopharmacology.

[34] Li Z, Zhang Y, Wang Z, Chen J, Fan J, Guan Y (2013). The role of BDNF, NTRK2 gene and their interaction in development of treatment-resistant depression: data from multicenter, prospective, longitudinal clinic practice. J Psychiatr Res.

[35] Benekareddy M, Mehrotra P, Kulkarni VA, Ramakrishnan P, Dias BG, Vaidya VA (2008). Antidepressant treatments regulate matrix metalloproteinases-2 and -9 (MMP-2/MMP-9) and tissue inhibitors of the metalloproteinases (TIMPs 1–4) in the adult rat hippocampus. Synapse.

[36] Integrative Biomedical Informatics Group GRIB/IMIM/UPF. Gene-disease association data retrieved from DisGeNET v6.0. 2020. 10.1093/nar.

[37] Naoi M, Maruyama W, Shamoto-Nagai M (2018). Type A monoamine oxidase and serotonin are coordinately involved in depressive disorders: from neurotransmitter imbalance to impaired neurogenesis. J Neural Transm.

[38] Lee BH, Kim YK (2010). The roles of BDNF in the pathophysiology of major depression and in antidepressant treatment. Psychiatry Investig.

[39] Holt LM, Hernandez RD, Pacheco NL, Torres Ceja B, Hossain M, Olsen ML Astrocyte morphogenesis is dependent on BDNF signaling via astrocytic TrkB.T1. *Elife* **8**. 10.7554/eLife.44667 (2019). 10.7554/eLife.44667 PMC6726422 31433295

[40] Stahlberg MA, Kügler S, Dean C Visualizing BDNF cell-to-cell transfer reveals astrocytes are the primary recipient of neuronal BDNF. bioRxiv. 255935; (2018).

[41] Field T, Diego M (2008). Cortisol: the culprit prenatal stress variable. Int J Neurosci.

