# Supplementary material for: Chronic cortisol differentially impacts stem cell-derived astrocytes from major depressive disorder patients
Source: Transl Psychiatry. 2021 Nov 30;11:608. doi: 10.1038/s41398-021-01733-9 (PMC8632962; doi:10.1038/s41398-021-01733-9)
Supplement: Supplementary file 15 — Supplementary Information [file 41398_2021_1733_MOESM15_ESM.docx]

**SUPPLEMENTARY INFORMATION**

**SUPPLEMENTARY METHODS**

**Western Blot**

Astrocytes were lysed with 1x RIPA buffer (Thermo Fisher) with added protease and phosphatase inhibitors (Thermo Fisher) for 5 min on ice. Samples were centrifuged at 15,000rcf for 15 min at 4°C and supernatant was transferred to a new tube. Western blots were performed with standard procedures and transferred to membrane using the iBlot gel transfer system (Life Technologies). Membrane was incubated in 5% milk for 30 min at room temperature before overnight incubation with primary antibodies in milk at 4°C (antibodies: mouse anti EAAT2, 1:1000, Santa Cruz Biotechnology sc-365634, Santa Cruz, CA; mouse anti GAPDH, 1:1000, Fitzgerald, Acton, MA). Next, membrane was incubated with secondary antibody conjugated to horseradish peroxidase for 1 hour at room temperature. Protein visualized with electrochemiluminescence detection kit (EMD Millipore) per manufacturer’s instructions.

**Cytokine Blot**

Astrocytes were treated with 10 ng/mL iL1-β for 5 hours in minimal media before culture media was collected and frozen. Human Cytokine Array Kit (R&D Systems; Minneapolis, MN) was performed according to manufacturer’s instructions with 1 mL of media. Scanned films were analyzed using ImageJ with intensity of background subtracted from intensity of each dot.

**Flow Cytometry**

Percentage of inflammation-active astrocytes was quantified as previously described^16^. Briefly, astrocytes were treated with protein transport blockers and 10 ng/mL iL1-β or vehicle for 5 hours. Suspended cells were immunostained for IL6 and IL8 and sorted on a BD FACSCanto II analyzer. Percentage of cells positive for stain was normalized to vehicle-treated samples via subtraction.

**Click-iT™ EdU Cell Proliferation Immunostain**

Click-iT™ EdU Cell Proliferation Kit (Invitrogen C10340) was used according to manufacturer’s instructions. Briefly, we incubated cells with 10µM EdU for 2 hours or 24 hours. We followed our immunostain protocol and applied the Click-iT reagent mix directly before incubating with secondary antibody. Primary antibodies for this experiment were Ki-67 (mouse; BD Pharmingen 550609) and Caspase 3 (rabbit; Cell Signaling 9661).

**SUPPLEMENTARY FIGURES**


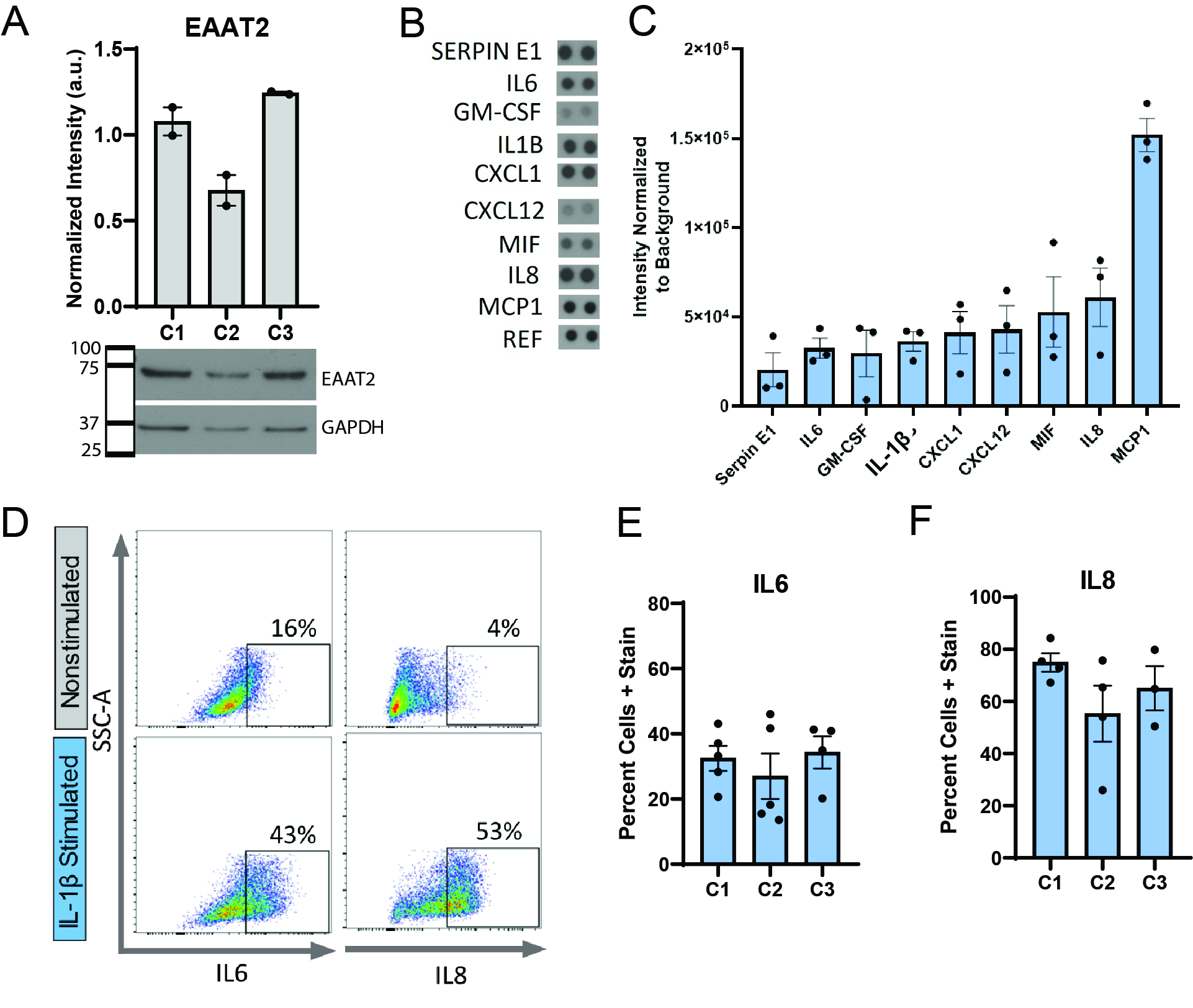


**Figure S1: iPSC-derived astrocytes differentiated in serum-free conditions express glutamate transporter and are capable of activation following iL1-β stimulation.**

(A) Quantified Western blot of glutamate transporter EAAT2 protein expression; mean ± SEM, n=2 replicates. (B) Representative images of cytokine blot measuring cytokine release in cell culture media following 5-hour stimulation with iL1-β. (C) Quantification of cytokine blot showing mean intensity ± SEM, n=3 individuals. (D) Representative FACs plots of nonstimulated and iL1-β-stimulated astrocytes producing cytokines IL6 and IL8. (E) Quantification of FACs analysis showing percentage of cells producing IL6 or (F) IL8 following stimulation normalized to vehicle by subtraction. For each individual, dots indicate technical replicates. Bars show mean ± SEM; (E) n=5 and (F) n=3 replicates for each individual.

­


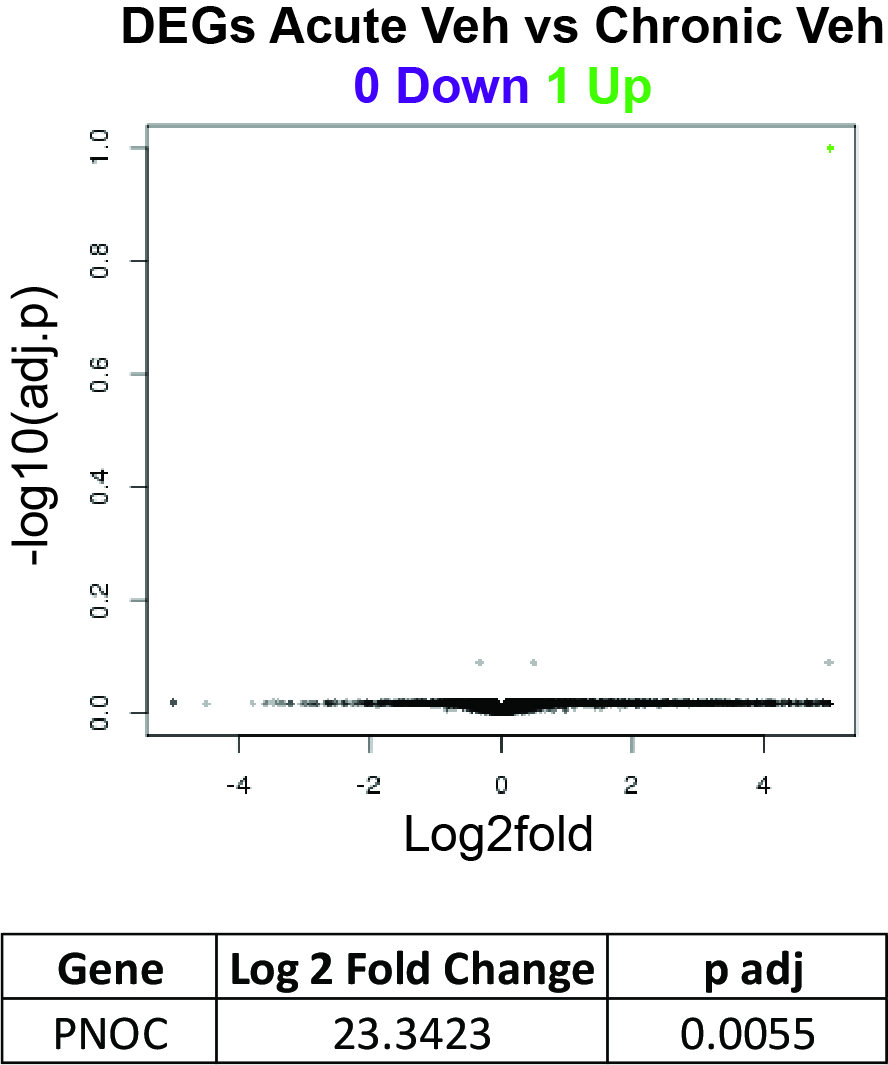


**Figure S2: Vehicle (Ethanol) comparison and overlapping gene sets in acute and chronic treatment.**

Volcano plot showing 1 DEG between acute and chronic vehicle treatment. Log2 Fold Change and p value listed below.

**
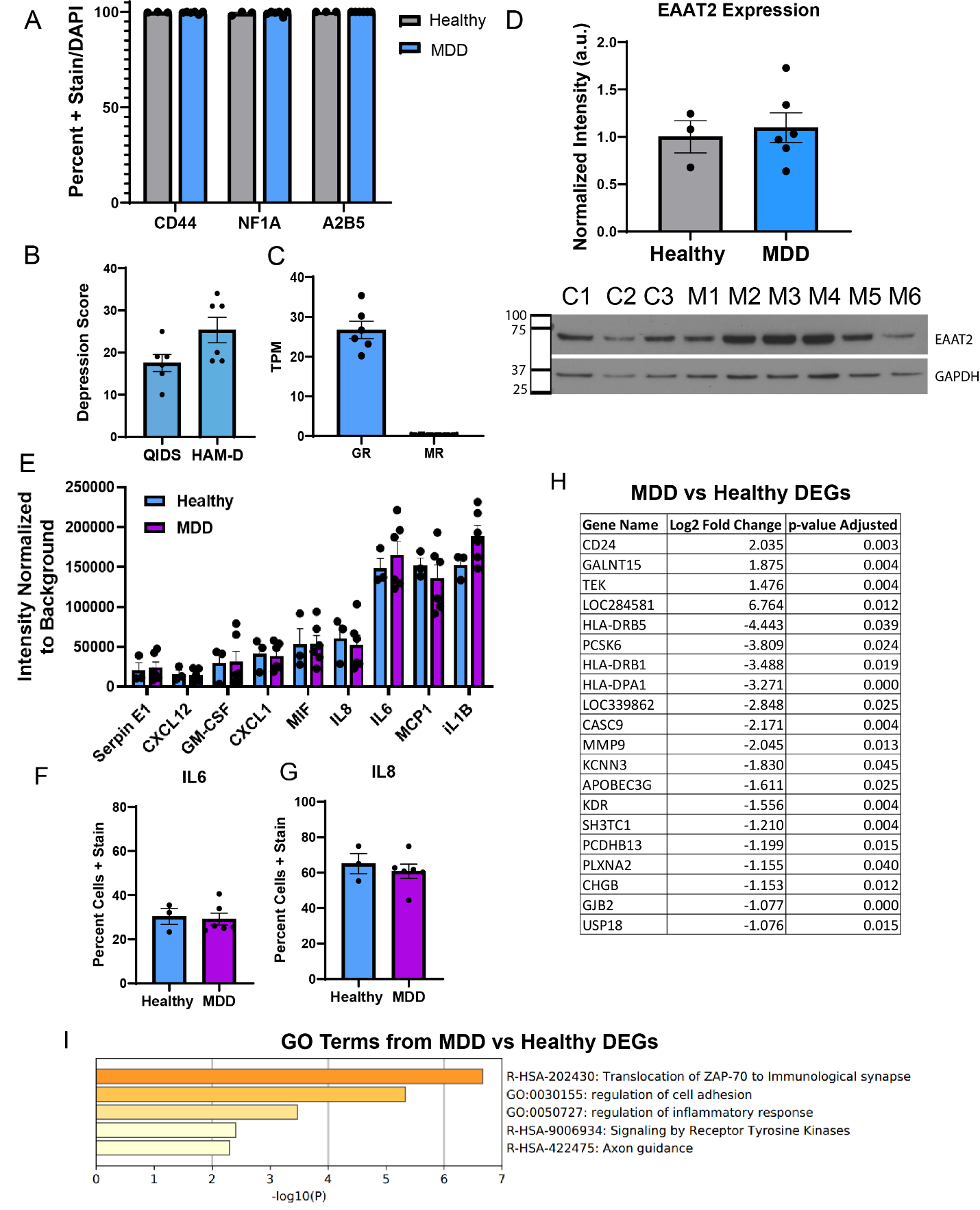
Figure S3: Astrocytes derived from MDD lines show similar gene expression and cellular function as healthy controls.**

(A) Quantified immunostain of early glial markers expressed in GPCs derived from MDD lines. Bars show mean ± SEM; n=3 control; n=6 MDD. (B) Depression scores of MDD patients quantified with QIDS and HAM-D clinical evaluation; bars represent mean ± SEM for n=6 individuals (each individual is represented by a dot). (C) TPM expression values from RNA-sequencing showing GR and MR expression in MDD lines under baseline conditions Bars show mean ± SEM; n=6. (D) Quantified Western blot EAAT2 protein expression. Bars show mean ± SEM for n=3 control; n=6 MDD; 2 replicates per line. Representative image of EAAT2 Western blot for all lines below. (E) Quantified cytokine blot array measuring cytokine release in cell culture media after 5-hour incubation with iL1-β. Bars show mean ± SEM; n=3 control; n=6 MDD. (F) Quantification of FACs analysis showing percentage of cells producing IL6 or (G) IL8 following stimulation normalized to vehicle by subtraction. Bars show mean ± SEM; n=3 control; n=6 MDD. (H) Whole transcriptome sequencing under baseline conditions identified 20 DEGs between healthy and MDD astrocytes. Log2fold change and adjusted p-value listed in table. (I) Gene ontology terms gathered from the list of 20 DEGs.


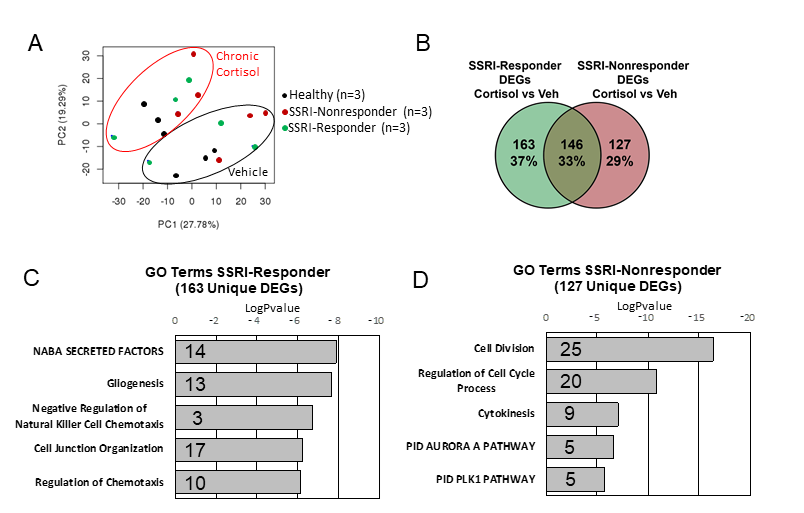


**Figure S4: Astrocyte gene expression following chronic cortisol treatment segregated by MDD patient response to SSRI antidepressants.**

(A) Principal component analysis of transcriptome sequencing data in all treated samples; black dots = healthy individuals, red dots = MDD individuals who are SSRI-Nonresponders, green dots = MDD individuals who are SSRI-Responders. (B) Venn Diagram comparison of DEGs identified in whole transcriptome sequencing unique to each subgroup and genes that appear in both lists after chronic cortisol treatment. (C) Top hits for gene ontology analysis of unique DEGs identified in SSRI-responders and (D) SSRI-Nonresponders. There were 209 DEGs in responders and 273 DEGs in nonresponders, with a majority of the same DEGs dysregulated in both groups (Sup Fig 4B and Sup Table 10-11). To better understand any unique response to chronic cortisol that may be associated with SSRI responsiveness, we performed GO analysis of the 163 unique DEGs for responders (Sup Fig 4C and Sup Table 12) and the 127 unique DEGs for nonresponders (Sup Fig 4D and Sup Table 13). Many of the GO terms were also top hits identified earlier in the analysis of unique chronic cortisol DEGs including cell division, chemotaxis, and gliogenesis. Interestingly, the SSRI-nonresponder top hits include *cell division, regulation of cell cycle process,* and *cytokinesis* which are not as significant in the responder list.


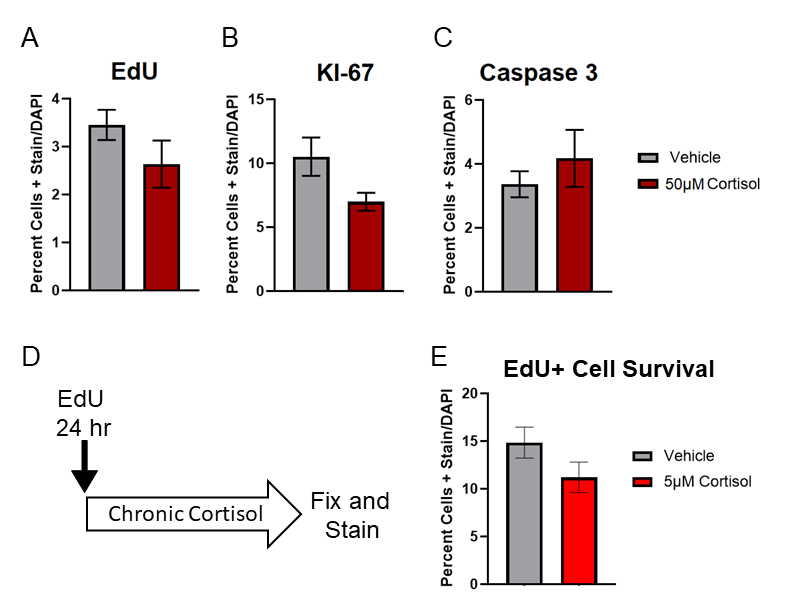


**Figure S5: Cell death and proliferation analysis following chronic cortisol.**

(A) Percent of astrocytes that are immunopositive for EdU after a 2 hour incubation with EdU following chronic cortisol or vehicle treatment (n=4 wells, one individual); bars represent mean ± SEM. (B) Percent of astrocytes that are immunopositive for KI-67 or (C) Caspase 3 following chronic cortisol or vehicle treatment (n=4 wells, one individual); bars represent mean ± SEM. (D) Schematic depicting “EdU+ Cell Survival” paradigm. Cells are incubated overnight with EdU followed by chronic cortisol or vehicle treatment. (E) Percent of astrocytes immunopositive for EdU following chronic cortisol (n=3 pictures, one individual); bars represent mean ± SEM.
